# Correction: Jung et al. miR-409-3p Regulates IFNG and p16 Signaling in the Human Blood of Aging-Related Hearing Loss. *Cells* 2024, 13, 1595

**DOI:** 10.3390/cells15080738

**Published:** 2026-04-21

**Authors:** Junseo Jung, Jeongmin Lee, Hyunsook Kang, Kyeongjin Park, Young Sun Kim, Jungho Ha, Seongjun So, Siung Sung, Jeong Hyeon Yun, Jeong Hun Jang, Seong Jun Choi, Yun-Hoon Choung

**Affiliations:** 1 Department of Otolaryngology-Head and Neck Surgery, Cheonan Hospital, College of Medicine, Soonchunhyang University, Cheonan 31151, Republic of Korea; ing2094@naver.com (J.J.); minn5454@naver.com (J.L.); ssook43111@gmail.com (H.K.); rudwls1289@naver.com (K.P.); 2 Department of Biomedical Science, Soonchunhyang University, Asan 31538, Republic of Korea; 3 Department of Otolaryngology, School of Medicine, Ajou University, Suwon 16499, Republic of Korea; jmyea@hanmail.net (Y.S.K.); jhflyflyfly@gmail.com (J.H.); thtjdwns123@gmail.com (S.S.); jhj@ajou.ac.kr (J.H.J.); 4 Department of Medical Sciences, Graduate School of Medicine, Ajou University, Suwon 16499, Republic of Korea; 5 Department of Biomedical Sciences, Ajou University Graduate School, Suwon 16499, Republic of Korea; ylem2s1u@gmail.com (S.S.); medicake@ajou.ac.kr (J.H.Y.)

## Affiliation Corrections

In the original publication [1], there were errors regarding the affiliations of the following authors: Siung Sung, Jeong Hyeon Yun and Yun-Hoon Choung. For Siung Sung and Jeong Hyeon Yun, affiliation 4 was replaced with affiliation 5: Department of Biomedical Sciences, Ajou University Graduate School, Suwon 16499, Republic of Korea. For Yun-Hoon Choung, in addition to affiliations 3 and 4, the following affiliation was added: Department of Biomedical Sciences, Ajou University Graduate School, Suwon 16499, Republic of Korea.

Also, there was a typo in author Siung Sung’s email address due to an oversight. This has now been corrected as well. In addition, Ms. Kang’s email address has been updated.

The authors state that the scientific conclusions are unaffected. The original publication has also been updated.
